# Assessment of Emotional Expressions after Full-Face Transplantation

**DOI:** 10.1155/2017/8789724

**Published:** 2017-06-21

**Authors:** Çağdaş Topçu, Hilmi Uysal, Ömer Özkan, Özlenen Özkan, Övünç Polat, Merve Bedeloğlu, Arzu Akgül, Ela Naz Döğer, Refik Sever, Nur Ebru Barçın, Kadriye Tombak, Ömer Halil Çolak

**Affiliations:** ^1^Faculty of Engineering, Department of Electrical-Electronics Engineering, Akdeniz University, Antalya, Turkey; ^2^Faculty of Medicine, Department of Neurology, Akdeniz University, Antalya, Turkey; ^3^Faculty of Medicine, Department of Plastic and Reconstructive Surgery, Akdeniz University, Antalya, Turkey; ^4^Faculty of Medicine, Department of Physical Medicine and Rehabilitation, Akdeniz University, Antalya, Turkey

## Abstract

We assessed clinical features as well as sensory and motor recoveries in 3 full-face transplantation patients. A frequency analysis was performed on facial surface electromyography data collected during 6 basic emotional expressions and 4 primary facial movements. Motor progress was assessed using the wavelet packet method by comparison against the mean results obtained from 10 healthy subjects. Analyses were conducted on 1 patient at approximately 1 year after face transplantation and at 2 years after transplantation in the remaining 2 patients. Motor recovery was observed following sensory recovery in all 3 patients; however, the 3 cases had different backgrounds and exhibited different degrees and rates of sensory and motor improvements after transplant. Wavelet packet energy was detected in all patients during emotional expressions and primary movements; however, there were fewer active channels during expressions in transplant patients compared to healthy individuals, and patterns of wavelet packet energy were different for each patient. Finally, high-frequency components were typically detected in patients during emotional expressions, but fewer channels demonstrated these high-frequency components in patients compared to healthy individuals. Our data suggest that the posttransplantation recovery of emotional facial expression requires neural plasticity.

## 1. Introduction

Facial anatomy not only affects functional aspects of daily life such as eating and speaking but also affects an individual's psychology and social relationships.

Unilateral and bilateral hand and arm transplantations have been performed safely and successfully around the world since 1998 [1]. More recently, surgeons have begun to optimize and implement partial- and complete-face transplantations, in part due to the failure of conventional approaches to facial reconstruction. In 2005, a partial-face transplantation was performed on a 38-year-old woman who had been bitten by a dog; this patient has since undergone additional surgical treatment [2]. Since 2005, a total of 28 partial- and full-face transplantations have been performed. Functional improvements (e.g., ability to eat, drink, speak, smell, and smile) after the operation were reported in all patients [3]. Over time, functional improvements in emotional expression were also reported in the first partial-face transplant case [4]. However, no study to date has comprehensively assessed the recovery of emotional expression after full-face transplant or evaluated modifications in the somatosensory cortex after face transplantation [5].

In this study, we assessed clinical features as well as motor and sensory improvements after full-face transplantation in 3 patients. Semmes-Weinstein's monofilament test (SWMT) was used to evaluate touch thresholds; a monofilament kit comprising 20 monofilaments with 4 different levels was employed to obtain normal values for various areas including the cervical dermatomes, soles, and palms [6]; however, normal values were not recorded for patients' faces so that each patient's progression could be examined separately. After surgery, we performed a frequency analysis on facial surface electromyography (sEMG) data collected during 6 basic emotional expressions and 4 primary facial movements. Motor progress was assessed using the wavelet packet method by comparison against healthy subjects. All patients had difficulty with emotional facial expressions, consistent with a previous work [7]. Motor recovery was slower than sensory improvements, also in agreement with a previous report [8]. However, as sensory and motor reinnervation progressed, so did the patients' abilities to demonstrate emotional expressions. These data inform the expected course of recovery and rehabilitation after full-face transplantation.

## 2. Materials and Methods

### 2.1. Patients

#### 2.1.1. Case 1

A 28-year-old male patient had lost his left eye, nose, a majority of the bilateral maxilla, and the bilateral anterior region of the mandible's angle due to an attempted suicide 4 years prior. The patient had a tracheostomy and was fed by nasogastric catheter. He underwent complete-face transplantation in July 2013. The infraorbital nerve was coapted, and the patient's own orbital and frontal branches were retained. The other inferior branches were splinted from the donor's facial trunk to the patient's trunk. Unlike cases 2 and 3, the maxilla and mandible were also transferred, and therefore, the infra-alveolar nerve was coapted to that of the donor. The palate and all muscles for facial expression were transferred. Skin incisions were placed frontally, inferior to the hairline, and bilaterally in the preauricular area, to 3 cm below the hyoid bone.

During the first year after surgery, no clear motor improvements were observed. However, primary facial movements had clearly developed by the end of the second year. In contrast, sensory recovery was observed from the 4th month after surgery.  Table 1 shows the SWMT results of case 1 across 20 consecutive months of observation.

#### 2.1.2. Case 2

The second case was a 37-year-old man whose face was completely burned when he was 3 years old.

When the patient was admitted to the polyclinic, both his eyelids exhibited ectropion and epiphora and there was a hairless ulcerated region in the parietal area. His right ear was missing and he was completely unable to use his facial muscles for expression. The patient underwent face transplantation in May 2012. The bilateral intraorbital, supraorbital, and mental nerves were coapted. The patient's bilateral facial nerve trunks were coapted to those of the donor; no other branches were coapted because the eyelids were also transferred on this patient. Different from cases 1 and 3, the right ear, hairy skin from the parietal area, and the bilateral lacrimal ducts were transferred to the patient. All muscles for facial expression were transplanted, but those for mastication were not. Skin incisions were made in the parietal region (in the hairy skin), 10 cm posterior to the hairline; posterior to the right ear; in the left preauricular area; and 3 cm below the hyoid bone on the neck.

Three months after surgery, the patient was able to distinguish touch and pain sensation. Two-point discrimination developed after the 5th month. Similar to case 1, facial agraphesthesia began to recover after the 6th month. Motor improvements were observed after the 8th month.

#### 2.1.3. Case 3

A 22-year-old patient had burns on his entire face due to exposure to boiling water at 7 months of age. He had no motor control over facial expression upon presentation to our plastic and reconstructive surgery polyclinic.

The patient underwent face transplantation in January of 2012 with the complete face of a 37-year-old donor excluding the eyelids. The infraorbital, supraorbital, and mental nerves were coapted. The lower branches were also coapted to the donor's facial nerve trunk. All muscles for facial expression were transplanted, but those for mastication were not. Skin incisions were made just below the hairline and in the bilateral preauricular area to 3 cm below the hyoid bone.

Approximately 3 months after surgery, the patient showed improvements in touch and pain sensation. Recovery of 2-point discrimination appeared at 6 months postsurgery. Needle EMG performed at 6 months postsurgery demonstrated reinnervation activity in the frontalis, orbicularis oculi, and oris muscles. The recovery of motor control began slowly during this period and became noticeable at 14 months postsurgery through actions such as eyebrow movement, closing his eyes, and showing his teeth. Although the patient did not have problems recognizing pictorial representations of emotional facial expressions, his own emotional expressions did not completely recover. Functional magnetic resonance imaging data reported in another study demonstrated interactions between cortical representations of the face and hand and cortical plasticity [9].

Surprisingly, in a regular check-up approximately 3 years postsurgery, the patient indicated that he felt sensation on the right side of his face when the fingers of his right hand were touched by a brush. This phenomenon was confirmed in later check-ups, and we suspected that it might have been due to progressive plastic changes in the cortical area representing the face and its proximity to that representing the hand. After the observation of improvements in basic skin sensation, we evaluated graphesthesia. The patient initially demonstrated agraphesthesia but showed improvement after the recovery of 2-point discrimination. The patient's level of localization and tactile sense have since shown improvement, especially on the cheeks.

For all 3 patients, thymoglobulin (1.25 mg/kg) and prednisolone (initiated at 1000 mg/day and decreased postsurgery) were administered during the surgery. At 7 days postsurgery, tacrolimus therapy (0.2 mg/kg, serum level 15–20 ng/mL) was initiated. Thymoglobulin was discontinued after the 10th day. Thereafter, treatment was continued with prednisone (20 mg/day), tacrolimus, and mycophenolate mofetil (2 g/day).

### 2.2. Data Recording and Processing

sEMG recordings were performed for the abovementioned 3 full-face transplantation patients and 10 healthy individuals. Selected healthy individuals were male (31 ± 5 years) to control for sex differences in facial movements. Recordings were made from 14 bipolar electrodes (diameter, 9 mm; interelectrode distance, 20 mm) using conductive and adhesive neurodiagnostic gel.

Subjects were asked to perform simple facial expressions (anger, fear, happiness, hate, surprise, and sadness) [10, 11], and spectral analyses were performed on the resulting sEMG data. While it can be difficult to discriminate between basic emotions and facial sEMG, it has generally been demonstrated to be a sensitive technique [12].

Each recording period included 2 s of activation and 3 s in a resting position. The PowerLab 35/15 recording system (sample rate, fs = 2000 Hz) was used for measurements. High-definition videos were also captured. For the analysis, we divided the face into 2 regions: the upper face, which was evaluated by channels 1–6, and the lower face, which was evaluated by channels 7–14.

For data processing, notch filters were initially applied to electrode signals in order to remove power line interference. A 6th-order Butterworth band-pass filter (3–450 Hz) was used to minimize motion artifacts and noise. Finally, signals were full-wave-rectified.

After filtering, signals were decomposed into frequency bands using the wavelet packet method. Wavelet packets are a generalization of the connection between multiresolution methods and wavelets [13]. This generalization is illustrated by

(1) $$\begin{array}{c} W_{m,j,n}\left(t\right)=2^{-m/2}W_{j}\left(2^{-m}t-n\right), \end{array}$$

where *j* ∈ *N* denotes the node index in each *m* level [14]. The root mean square value of the decomposition components can be defined as

(2) $$\begin{array}{c} w_{rms,m,j}=\sqrt{\frac{1}{N}\sum {\left|w_{m,j}\left(r\right)\right|}^{2}}. \end{array}$$

The energy of a wavelet packet is related to the frequency characteristic of sEMG signal. Total wavelet packet energy for each node can be calculated as

(3) $$\begin{array}{c} E_{tot}=\overset{2^{M}-1}{\underset{j=0}{\sum }}{\left|w_{rms,m,j}\right|}^{2}. \end{array}$$

Moreover, the frequency value of a wavelet packet can be calculated as

(4) $$\begin{array}{c} f_{m}=\frac{\left(n+1\right)f_{s}}{2^{m+1}},m=1,follows, \end{array}$$

where *f*_s_ is the sampling frequency and *f*_*m*_ is the frequency in the *m*th level. An interval of *n* is indicated as *n* = 0, 1,…, 2^*m*^ − 1 for the wavelet packet [15, 16]. Seventh-level wavelet packet decomposition was used to calculate wavelet packet energy. Daubechies 5 was chosen as a mother wavelet.

## 3. Results and Discussion

### 3.1. Primary Facial Movements

Overall, we observed sEMG activity during basic movements for each electrode position in all patients and healthy subjects.

For the lip funneler movement [17], case 3 showed a similar pattern to that of healthy subjects, including high-frequency components. All healthy subjects and patients exhibited high-frequency components on sEMG for this movement.

For the lid tightener movement, cases 1 and 3 showed patterns that were similar to those of healthy subjects. The ratio between the maximum wavelet packet energy value for case 1 and the mean value for healthy subjects was 0.173. All healthy subjects and patients exhibited high-frequency components on sEMG for this movement.

**Figure 1:**
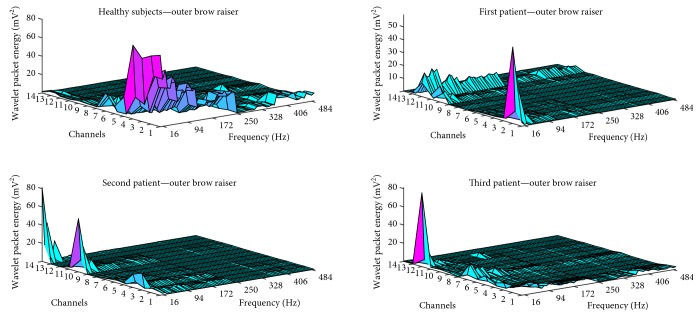
Wavelet packet energy data for the outer brow raiser movement.

For the lip suck and outer brow raiser facial movements, all patients showed patterns that were different from those of healthy subjects. All healthy subjects and patients exhibited high-frequency components on sEMG data for these movements. Wavelet packet energy data for the outer brow raiser movement in all subjects are illustrated in Figure 1.

### 3.2. Emotional Expressions

**Figure 2:**
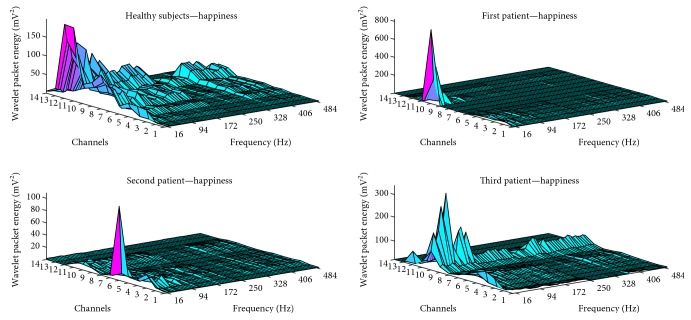
Wavelet packet energy data for the happiness facial expression.

During the happiness facial expression, case 3 showed a similar pattern to that of healthy subjects, including high-frequency components. However, cases 1 and 2 did not demonstrate high-frequency components during this expression. In the patient group, significant activity and amplitude differences were detected in the lower face. Wavelet packet energy data for the happiness facial expression in all subjects are illustrated in Figure 2.

**Figure 3:**
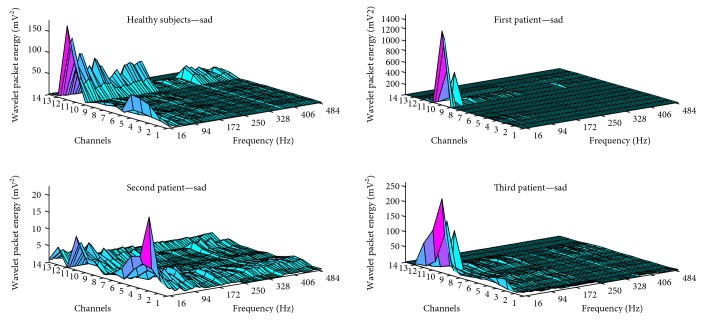
Wavelet packet energy data for the sadness facial expression.

During the sadness facial expression, case 3 showed a similar pattern of activity to that of healthy subjects. However, only healthy subjects and case 2 demonstrated high-frequency components during this expression. Wavelet packet energy values recorded for case 1 were the highest for the sadness facial expression. In cases 1 and 3, distinct spectral activity was observed, whereas unique activity in the lower face region was identified for case 2. Wavelet packet energy data for the sadness facial expression in all subjects are illustrated in Figure 3.

During the anger facial expression, case 2 demonstrated a similar pattern of activity to that of healthy subjects, including high-frequency components. However, cases 1 and 3 showed different patterns of activity; wavelet packet energy in cases 1 and 3 was much greater than that in healthy subjects in low frequencies, while these patients' high-frequency components demonstrated nearly 0 energy values. Moreover, cases 1 and 3 predominantly demonstrated activity in the lower face region during this expression.

During the fear facial expression, case 1 had a similar pattern to that of healthy subjects. All patients and subjects exhibited high-frequency components except for case 3. In cases 2 and 3, activity was primarily recorded in the upper face region.

During the hate/disgust facial expression, case 3 demonstrated a similar pattern of activity to that of healthy subjects. All patients and subjects exhibited high-frequency components except for case 1. In cases 1 and 3, activity and amplitude differences were predominantly observed in the lower face region.

During the surprised facial expression, case 2 showed a similar pattern of activity to that of healthy subjects, including high-frequency components. In case 3, activity was observed in the upper face, whereas in cases 1 and 2, activity was observed in the lower face during this expression. Active electrodes for all frequency regions, maximum wavelet packet energy values, the rate of maximum values, and peak channel numbers for all emotional expressions are listed in Table 2.

## 4. Conclusions

The 3 full-face transplantation patients in this study demonstrated acceptable levels of sensory and motor recoveries postsurgery. Although the patients demonstrated innervation of the facial muscles and successfully performed primary facial movements, patients had clear impairments in emotional expression. Our comparison between patients and healthy subjects can be used to develop new rehabilitation strategies after partial- or full-face transplantation.

Although all 3 patients in this study underwent a full-face transplantation procedure, there were some minor differences in their specific surgical procedures due to variations in injury etiology and localization. Despite these differences, touch and pain sensations were largely recovered within 3-4 months postsurgery, while 2-point discrimination returned after 5-6 months. Rates of improvement were slower than anticipated, but improvements continued through the first year. Prior to the first face transplantations, the restoration of normal facial skin was not considered to be possible. However, an increasing number of face transplantations have demonstrated sensory restoration within the first 3 months and the restoration of warm and cold sensation accompanied by tactile improvements and 2-point discrimination at approximately 8 months postsurgery [3]. Of note, sensory recovery appears to show no relationship with the techniques used for treatment. Indeed, comparable sensory improvements were observed after use of a simple transplantation method [18].

Unlike sensory recovery, functional motor recovery was initially noticed at 3-4 months postsurgery and clearly observable after 12–14 months. Other reports have indicated motor improvements beginning 3–6 months postsurgery and that continue through the end of the first year [15]. Functional recoveries such as eating, drinking, and speaking show a strong correlation with motor recovery in these patients.

Difficulty with facial emotional expressions after full-face transplantation has not yet been characterized in the literature. In this study, improvements in facial emotional expression were analyzed alongside functional recovery, evaluated at 1 year posttransplantation in 1 patient (case 2) and at 2 years posttransplantation in the remaining 2 patients (cases 1 and 3). In these 3 cases, we observed an initial “mask-like” expression that partially but incompletely recovered; however, the patients themselves did not recognize any problems with emotional expression. Indeed, the frequency and spatial distributions of sEMG signal were significantly different between our 3 patients and normal individuals. We think that this data highlights a need for novel rehabilitation strategies after partial- or full-face transplantation. Furthermore, although our data are largely consistent with previously established patterns of improvement in primary sensory and motor modalities after face transplantation, we think that our complete data are important for appreciating aspects of the recovery processes that require plasticity, such as the expression of emotion, which requires the cooperation of high-level cortical control and complex peripheral sensory and motor organization. Standardizing protocols for observation after full-face transplantation will enable the useful comparison of data between patients. These observation protocols should not only evaluate primary sensory and motor modalities but also include assessments of cognitive and higher-level cortical functions such as emotional expression.

## Supplementary Material

Supplementary Appendix. Figure S1: Semmes Weinstein's monofilament test results for case 3. Figure S2: Positions and channel numbers of facial electromyography electrodes. Figure S3: Wavelet packet energy data for the anger facial expression. Figure S4: Wavelet packet energy data for the fear facial expression. Figure S5: Wavelet packet energy data for the hateful/disgust facial expression. Figure S6: Wavelet packet energy data for the surprise facial expression. Table S1: Semmes Weinstein's monofilament test results for case 2 across 27 consecutive months.

## Figures and Tables

**Table 1:** Semmes-Weinstein's monofilament test results for case 1 across 20 consecutive months.

Case 1	Touch		Localization	
	Right	Left	Right	Left
Forehead	2.83	2.83	3.61	3.61
Above eyebrows	3.61	3.61	3.61	4.31
Eyelid	3.61	3.61	4.31	4.31
Eyebrow midpoint	3.61	ND	4.56	ND
Nose	2.83	2.83	3.61	3.61
Upper lip	3.61	ND	3.61	ND
Lower lip	3.61	ND	3.61	ND
Chin	3.61	3.61	4.31	4.31
Below ear	ND	ND	ND	ND
Cheek	2.83	2.83	3.61	3.61

ND: not determined.

**Table 2:** Active electrodes in low- and high-frequency bands, maximum wavelet packet energy values, the rate of maximum values, and peak values for emotional expressions in all subjects.

Facial expression	Subject	Active electrodes at low frequencies	Max value of WPE (mV^2^)	Rate of max values	Active electrodes at high frequencies
Anger	Case 3	5, 10, 12 (7.8125–62.50 Hz)	1256	15.90	
	Case 2	2, 3, 4, 5, 6, 9, 11 (7.8125–242.1875 Hz)	32	0.41	3, 4, 9, 11 (304.6875–500 Hz)
	Case 1	2, 10, 12 (7.8125–93.75 Hz)	1563	19.79	
	Healthy subjects	1, 2, 3, 4, 5, 6, 7, 8, 11, 12, 13 (7.8125–242.1875 Hz)	79	1	3, 4, 7, 11, 12 (375–500 Hz)

Fear	Case 3	3, 4, 5, 6, 7, 8, 11, 12 (7.8125–242.1875 Hz)	308	2.27	6 (382.8125–500 Hz)
	Case 2	1, 2, 3, 4, 5, 6, 10, 11 (7.8125–242.1875 Hz)	23	0.17	
	Case 1	2, 3 (7.8125–117.1875 Hz)	127	0.94	10, 12 (375–500 Hz)
		10, 12 (15,625–242,1875 Hz)			
	Healthy subjects	1, 2, 3, 4, 5, 6, 7, 8, 9, 11, 12 (7.8125–242.1875 Hz)	135	1	11, 12 (312.5–500 Hz)

Happiness	Case 3	3, 4, 5, 6, 9, 10, 11, 12 (7.8125–242.1875 Hz)	402	2.03	10 (7.8125–500 Hz) 3, 4, 5, 6 (312.5–500 Hz)
	Case 2	3, 4, 6, 9, 10, 11 (7.8125–242.1875 Hz)	121	0.61	10, 11 (375–500 Hz)
	Case 1	2, 3, 4, 5, 10 (7.8125–242.1875 Hz)	825	4.16	
	Healthy subjects	3, 4, 5, 6, 7, 8, 9, 10, 11, 12 (7.8125–242.1875 Hz)	198	1	3, 4, 5, 6, 7, 8, 9, 10, 11, 12 (375–500 Hz)

Hate	Case 3	1, 3, 4, 5, 6, 7, 9, 10, 11, 12 (7.8125–242.1875 Hz)	311	2.09	9, 10, (11312.5–500 Hz)
	Case 2	3, 4, 5, 6, 10, 11 (7.8125–242.1875 Hz)	18	0.12	11 (375–500 Hz)
	Case 1	10 (7.8125–242.1875 Hz)	2309	15.49	
	Healthy subjects	3, 4, 5, 7, 8, 11, 12 (7.8125–242.1875 Hz)	149	1	8, 11, 12 (375–500 Hz)

Surprise	Case 3	3, 4, 5, 8, 12, 13 (7.8125–242.1875 Hz)	275	5.73	
	Case 2	2, 3, 4, 10, 11 (7.8125–242.1875 Hz)	8	0.16	10, 11 (375–500 Hz)
	Case 1	2, 10, 12 (7.8125–242.1875 Hz)	696	14.53	
	Healthy subjects	1, 2, 3, 4, 5, 7, 8, 11, 12 (7.8125–242.1875 Hz)	48	1	1, 2, 11, 12 (375–500 Hz)

Sadness	Case 3	3, 4, 5, 6, 9, 10, 11, 12 (7.8125–242.1875 Hz)	238	1.36	9, 10, 11 (375–500 Hz)
	Case 2	2, 3, 4, 5, 11, 13 (7.8125–242.1875 Hz)	27	0.15	2, 3, 4, 10, 11, 13 (375–500 Hz)
	Case 1	4, 10 (7.8125–242.1875 Hz)	1686	9.61	4, 10 (304.6875–500 Hz)
	Healthy subjects	1, 2, 3, 4, 5, 6, 7, 8, 11, 12 (7.8125–242.1875 Hz)	175	1	1, 2, 3, 4, 5, 6, 7, 8, 11, 12 (375–500 Hz)

## Abbreviations

sEMG: Surface electromyography
SWMT: Semmes-Weinstein's monofilament test.
