# Supplementary material for: Assessment of Emotional Expressions after Full-Face Transplantation
Source: Neural Plast. 2017 Jun 21;2017:8789724. doi: 10.1155/2017/8789724 (PMC5499241; doi:10.1155/2017/8789724)

Table e-1. SWMT results of second patient from 27 consecutive months

| Case 2 | **Touch** | | **Localisation** | |
| --- | --- | --- | --- | --- |
|  | Right | Left | Right | Left |
| Forehead | 2·83 | 2·83 | 2·83 | 2·83 |
| Above Eyebrows | 2·83 | 2·83 | 3·61 | 4·31 |
| Eyelid | 2·83 | 2·83 | 3·61 | 4·31 |
| Eyebrow midpoint | 2·83 | ND | 4·31 | ND |
| Nose | 2·83 | 2·83 | 4·31 | 4·31 |
| Upper lip | 2·83 | ND | 3·61 | ND |
| Lower lip | 2·83 | ND | 3·61 | ND |
| Chin | 2·83 | 3·61 | 4·31 | 4·31 |
| Below Ear | 3·61 | 3·61 | 3·61 | 3·61 |
| Cheek | 2·83 | 3·61 | 4·31 | 4·31 |

(ND: Not determined)

Supplemental data figure e-1


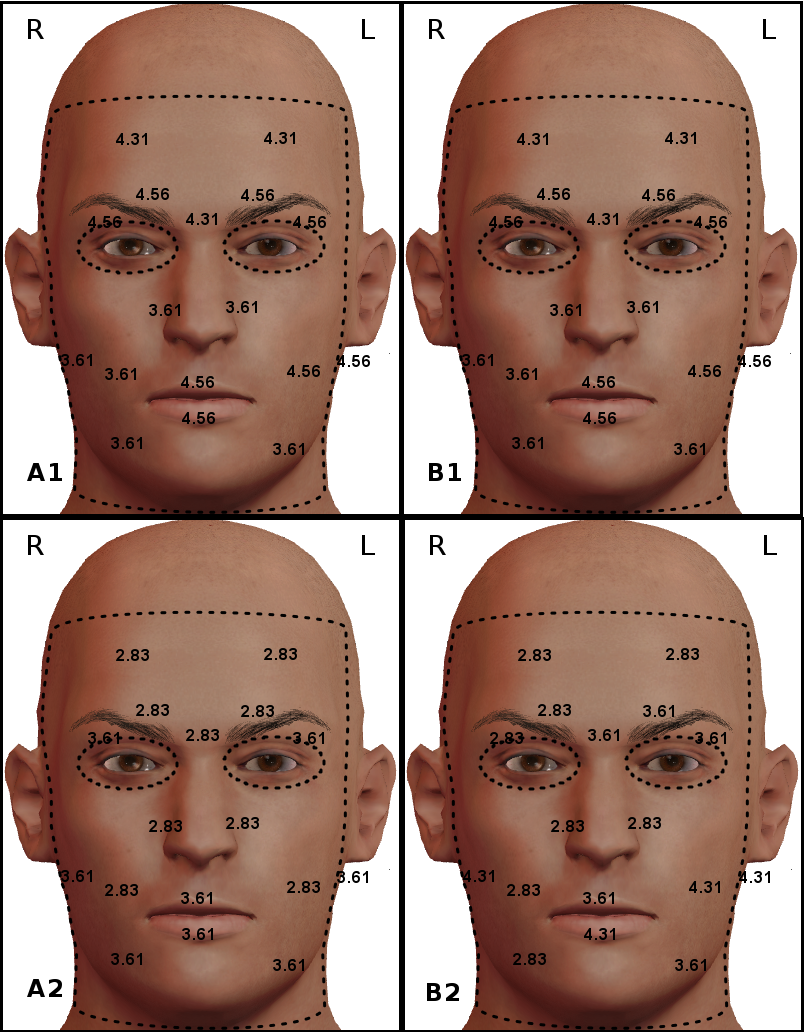


Supplemental data figure e-2


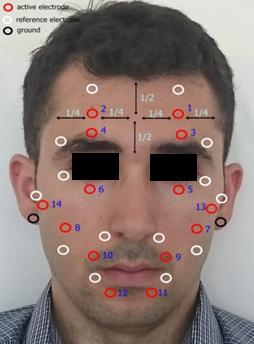


Supplemental data figure e-3


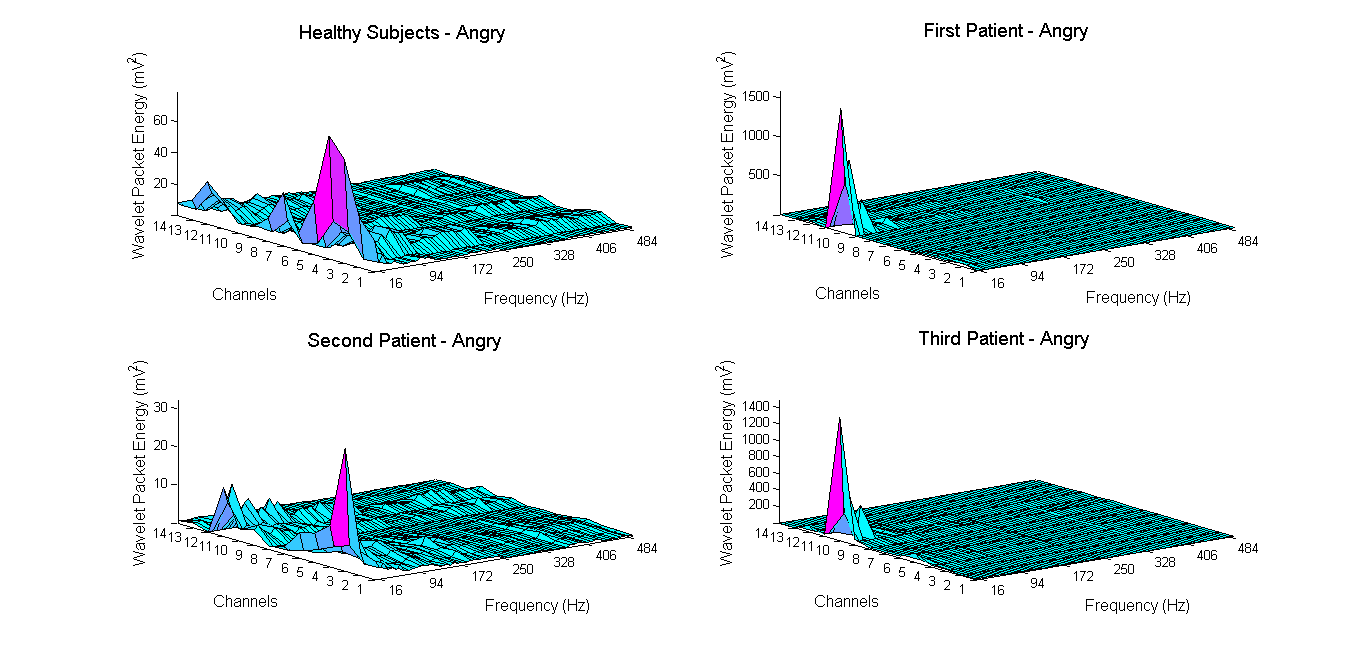


Supplemental data figure e-4


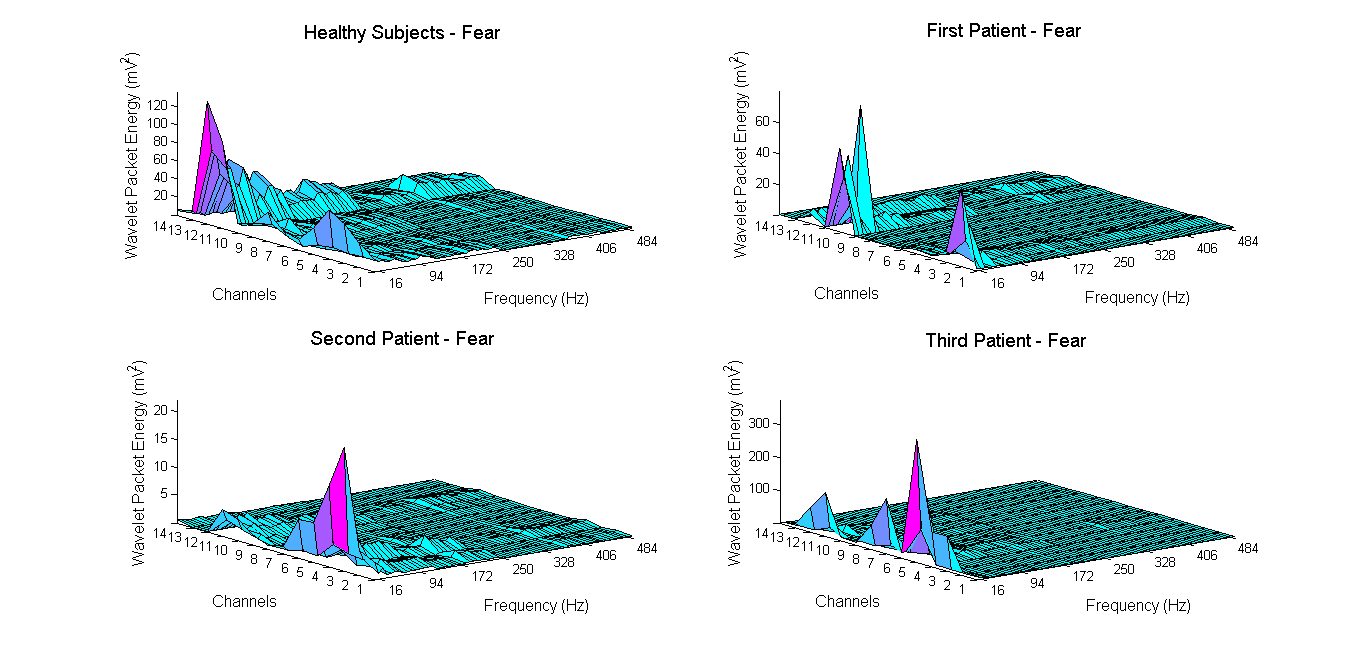


Supplemental data figure e-5


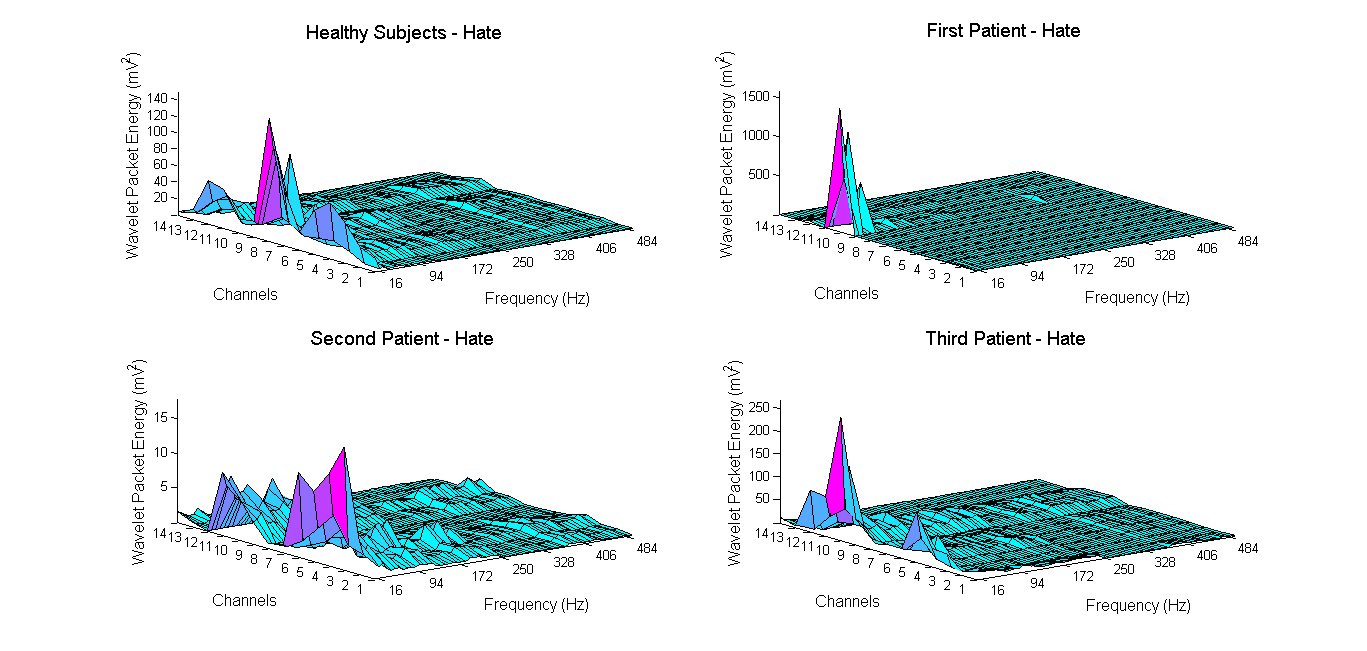


Supplemental data figure e-6


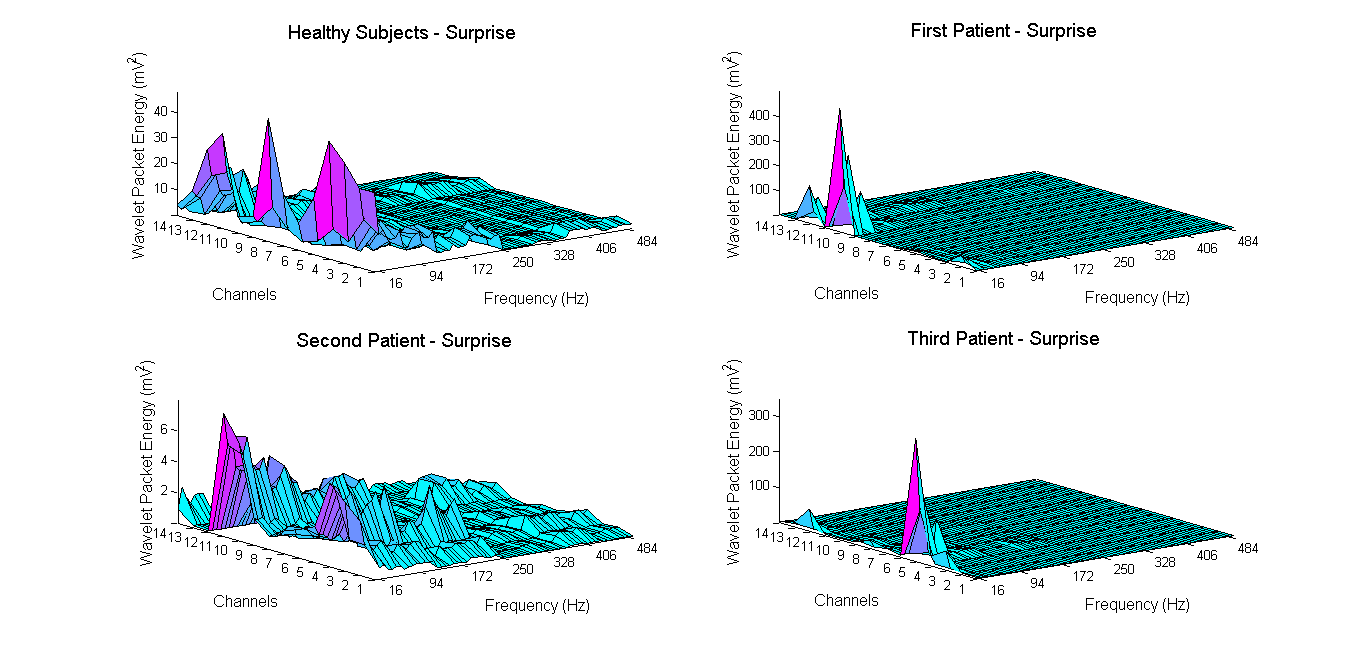

Supplement: Supplementary file 1 — Supplementary Appendix. Figure S1: Semmes Weinstein's monofilament test results for case 3. Figure S2: Positions and channel numbers of facial electromyography electrodes. Figure S3: Wavelet packet energy data for the anger facial expression. Figure S4: Wavelet packet energy data for the fear facial expression. Figure S5: Wavelet packet energy data for the hateful/disgust facial expression. Figure S6: Wavelet packet energy data for the surprise facial expression. Table S1: Semmes Weinstein's monofilament test results for case 2 across 27 consecutive months. [file 8789724.f1.docx]
